# Untargeted Metabolomics to Harness Ideal Protein Concept and Mitigate Environmental Impact in Rabbit Models

**DOI:** 10.3390/ijms26136047

**Published:** 2025-06-24

**Authors:** Pablo Jesús Marín-García, Jorge Mateo-López, César Cortés-García, Lola Llobat, Alejandro Huertas-Herrera, Mónica Toro-Manríquez, María Cambra-López, Juan José Pascual, Mette Skou Hedemann

**Affiliations:** 1 Department of Animal Production and Health, Veterinary Public Health and Food Science and Technology (PASAPTA), Facultad de Veterinaria, Universidad Cardenal Herrera-CEU, CEU Universities, 46113 Valencia, Spain; cesar.cortesgarcia@uchceu.es (C.C.-G.); maria.llobatbordes@uchceu.es (L.L.); 2 Institute for Animal Science and Technology, Universitat Politècnica de València, Camino de Vera s/n, 46022 Valencia, Spain; jmatlop@etsiamn.upv.es (J.M.-L.); macamlo@upvnet.es (M.C.-L.); jupascu@dca.upv.es (J.J.P.); 3 Centro de Investigación en Ecosistemas de la Patagonia (CIEP), Camino Baguales s/n Km 4.7, Coyhaique 5951601, Chile; alejandro.huertas@ciep.cl (A.H.-H.); monica.toro@ciep.cl (M.T.-M.); 4 Department of Animal and Veterinary Sciences, Aarhus University, Blichers Alle 20, DK-8830 Tjele, Denmark; mette.hedemann@anivet.au.dk

**Keywords:** pseudourine, citric acid, uridine, pantothenic acid, lysophosphatidylcholines

## Abstract

Environmental pollution remains a significant challenge in animal production. The “ideal protein” concept refers to an amino acid profile that precisely meets the animal’s nutritional requirements, optimizing nutrient utilization and minimizing waste excretion. This study applied untargeted metabolomics to explore metabolic changes induced by limiting AA. Two experimental diets were used in 47-day-old growing rabbits: Met+ (with a methionine level balanced to its optimal utilization) and Met− (with a methionine level that was clearly limiting). A total of 68 blood samples were taken for untargeted metabolomics analysis and 88 were taken for targeted plasmatic urea nitrogen analysis, collected at 08:00 (in ad libitum feeding animals) and 21:00 (after a feeding event in 10 h fasting animals). Our results revealed that both sampling time and diet (at each time point) exerted a significant modulatory influence on the metabolome. Interestingly, the difference between the metabolomes obtained with the different diets was less pronounced at 08:00, likely due to the caecotrophy effect, compared to 21:00, when higher intake and lower caecotrophy frequency were observed. This study identifies pseudourine, citric acid, pantothenic acid, and enterolactone sulfate as promising metabolites that could be targeted in order to refine the ideal protein concept, thus improving nutrient efficiency and reducing the environmental impact of animal production.

## 1. Introduction

Livestock significantly contributes to global nitrogen (N) excretion. In 2011, for example, the European Union (EU) produced 9.2 megatons of nitrogen in manure [1]. This manure leads to the emission of ammonia, nitrous oxide, and nitrogen oxides, among other compounds, which have been identified as contributing factors to environmental impacts such as eutrophication and greenhouse gas emissions [2,3]. In addition to this direct pollution, the contamination resulting from inefficient protein nutrition further increases the environmental impact, highlighting the importance of studying the amino acid (AA) profile of animal diets. An inadequate AA supply reduces animal performance, whereas an oversupply increases costs and leads to excessive nitrogen excretion, with the potential to harm the environment [4,5,6]. An imbalanced AA diet compels animals to catabolize excess AA, resulting in an energetic cost and ATP expenditure proportional to the amount of nitrogen excreted [7]. In summary, the precise alignment of the AA content of the diet with the nutritional requirements of the animals not only improves productivity but also assists in the mitigation of environmental pollution [8].

The concept of ideal protein can be defined as the AA profile of the diet that exactly matches the animal’s requirements. This AA combination optimizes protein utilization, thereby ensuring maximal retention and minimal excretion [5,6]. Achieving the ideal protein formulation requires a comprehensive understanding of the precise nutritional requirements tailored to each animal, in accordance with the dynamic principles of precision nutrition [9]. One of the main challenges in implementing this concept pertains to the dynamic determination of nutritional requirements. In this context, identifying biomarkers that exhibit rapid changes in response to AA balance may offer significant value.

The search for biomarkers, defined as objectively measurable characteristics, has already been applied to studies on dietary patterns [10] and dietary composition [11,12]. Some metabolites, such as plasma urea nitrogen (PUN), which is defined as the amount of circulating urea in the bloodstream, have been identified as biomarkers for the protein-rich diets of growing rabbits [8,13,14,15]. However, the advent of metabolomics has precipitated a paradigm shift within this domain. It has emerged as a powerful tool for investigating the metabolome of individuals and is proving to be valuable in the field of AA nutrition [16]. This holistic analysis of the subject’s metabolite profile enables a deeper comprehension of specific metabolic processes and, recently, this methodology has been recently integrated into precision nutrition for livestock species [17,18].

One of the typically limiting AA in growing rabbits is methionine, which is commonly grouped with cysteine as sulfur-containing AA. In this work, we refer to this group as ‘Met’. The prevailing nutritional recommendations for these sulfur AAs in growing rabbits were considered to be well-established and corresponded to a quantity of 5.8 g/kg dry matter (DM). However, research findings have indicated that there exists a higher nutritional requirement for Met [19,20,21].

The present study hypothesizes that short-term dietary changes—specifically those caused by a deficiency in a key essential amino acid—can lead to metabolomic alterations that may reveal biomarkers. These biomarkers could support the application of the ideal protein concept by helping the monitoring and adjustment of amino acid balance in diets, thereby reducing nitrogen excretion and environmental pollution. The objective of this study was to understand the effect of diet, feeding systems, and their interactions on metabolic phenotypes, and to identify potential biomarkers that could be used in the efforts to reduce pollution.

## 2. Results

Average daily weight gain (ADG; on av. 44.3 ± 0.68 g/d, between 28 and 63 d of age) was recorded to monitor rabbit health. No significant differences were observed between the diets (Diet Met+: 44.2 ± 1.10 g/d vs. Diet Met−: 44.3 ± 1.04 g/d; *p* = 0.9411). PUN levels were significantly different according to the sampling time (08:00: 11.06 ± 0.65 mg/dL vs. 21:00: 17.47 ± 0.65 mg/dL; *p* < 0.001). However, PUN levels were not significantly affected by the diet (Diet Met+: 13.57 ± 0.77 mg/dL vs. Diet Met−: 15.04 ± 0.85 mg/dL; *p* = 0.1992). When evaluating the diet x sampling time interaction effect, PUN was not affected by the diet at 8:00 (Met+: 11.32 ± 0.84^a^ mg/dL Met−: 10.76 ± 0.65^a^ mg/dL), but diet does affect at 21:00 (Met+: 15.91 ± 0.86^b^ mg/dL; Met−: 19.34 ± 0.65^c^ mg/dL; *p* < 0.001).

**Figure 1:**
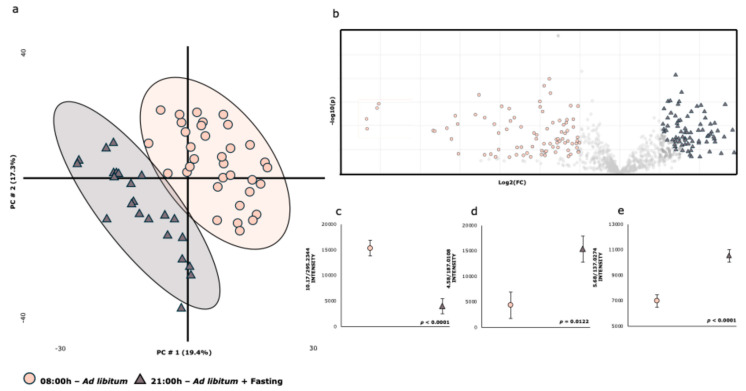
**Summary of the results regarding the impact of sampling time on the metabolome.** (**a**) PLS-DA score plot of the plasma metabolome. (**b**) Volcano plot showing differentially significant metabolites between two experimental sampling times (two-sided Wilcoxon rank tests with the value adjusted by false discovery rate, FDR < 0.05) are shown. (**c**–**e**) Effect of the sampling time on the intensity of metabolites 10.17/295.2344 (**c**), 4.58/187.0108 (**d**), and 5.68/137.0274 (**e**). The colors and shapes correspond to the two sampling times (R^2^ = 0.82596 and Q^2^ = 0.64514, respectively).

**Figure 2:**
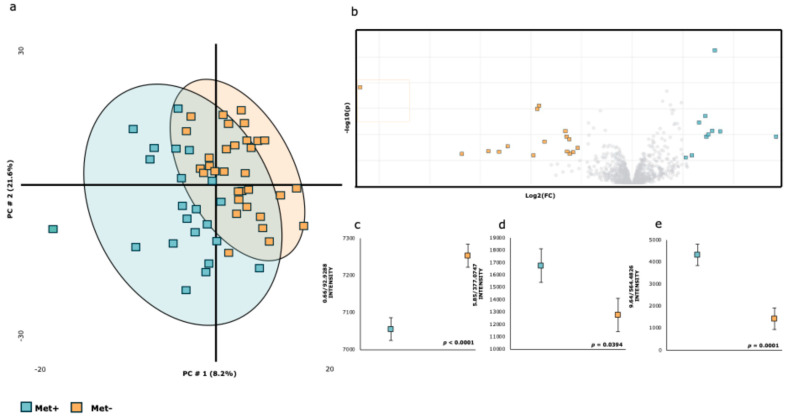
**Summary of the results regarding the impact of the experimental diet at 08:00 h on the metabolome.** (**a**) PLS-DA score plot of the plasma metabolome. (**b**) Volcano plot showing significantly different metabolites between the two experimental diets at 08:00 h (two-sided Wilcoxon rank test with the value adjusted by the false discovery rate, FDR < 0.05) are shown. (**c**–**e**) Effect of the experimental diet at 08:00 h on the intensity of metabolites 0.66/92.9288 (**c**), 5.85/377.0747 (**d**), and 9.64/564.4826 (**e**). The colors and shapes correspond to the two experimental diets (R^2^ = 0.92911 and Q^2^ = 0.3857, respectively).

Figure 1 shows the effect of sampling time on the PLS-DA score plot of the data obtained from the non-targeted metabolomics analysis. Figure 1a shows the first two principal components from PLS-DA, which explain 37% of the total variance, effectively differentiating the experimental diets with minimal group overlap. The volcano plot (Figure 1b) identifies the key metabolites contributing to this differentiation, which are highlighted. Finally, Figure 2c–e show the levels of three specific metabolic features (9-Hydroxylinoleic acid (1c), *p*-cresol sulfate (1d), and 4-Hydroxybenzoic acid (2e)). Table 1 presents the list of plasma metabolites that vary with sampling time, with 19 out of the 23 identified metabolites showing significant differences.

The model including diet as the primary factor exhibited signs of overfitting, making its outputs unreliable and therefore unsuitable for interpretation or reporting. This is the main reason why it has not been performed.

**Figure 3:**
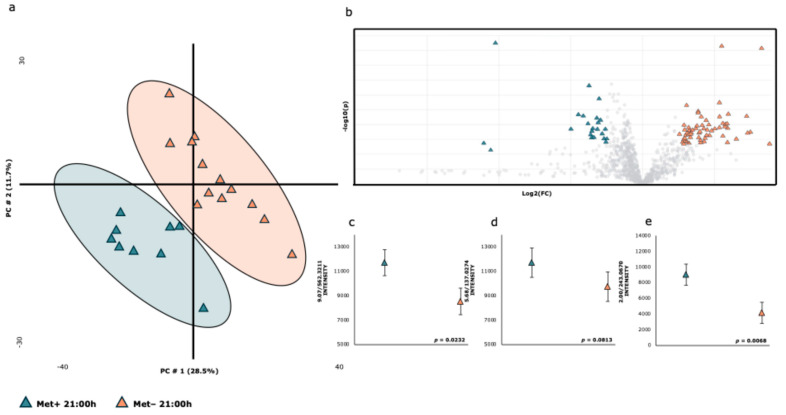
**Summary of the results regarding the impact of the experimental diet at 21:00 h on the metabolome.** (**a**) PLS-DA score plot of the plasma metabolome. (**b**) Volcano plot showing significantly different metabolites between the two experimental diets at 21:00 h (two-sided Wilcoxon rank test with the value adjusted by the false discovery rate, FDR < 0.05) are shown. (**c**–**e**) Effect of the experimental diet at 21:00 h on the intensity of metabolites 9.07/562.3211 (**c**), 5.68/137.0274 (**d**), and 2.00/243.0670 (**e**). The colors and shapes correspond to the two experimental diets (R^2^ = 0.92388 and Q^2^ = 0. 72715, respectively).

Figure 2 shows the effect of the experimental diet on the plasma metabolome of samples collected at 08:00. Figure 3a shows the PLS-DA score plot, where the first two principal components explained 37% of the variance and allowed partial separation between the experimental diets, although some overlap remained. The volcano plot (Figure 2b) identifies the most discriminating metabolites, while Figure 2c–e show the levels of specific metabolic features (Chloroacetic acid (2c), Enterolactone sulfate (2d), LysoPC (2e)). Table 2 shows the list of plasma metabolites that change with the experimental diets at 08:00, with 3 out of the 23 identified metabolites showing significant differences.

Figure 3 represents the effect of the experimental diet on the plasma metabolome of samples collected at 21:00. Figure shows a clear differentiation between the groups on the first two principal components, which explained 40% of the variance in the dataset and allowed for clear differentiation, with no group overlap observed. The volcano plot (Figure 3b) identifies discriminative metabolites, while Figure 3c–e show the levels of metabolic features (LysoPC(18:3/0.0) (4c), 4-Hydroxybenzoic acid (3d), and uridine (3e)). Table 3 shows the list of plasma metabolites that were shifted with the experimental diets at 21:00, with 5 out of the 23 identified metabolites showing significant differences.

**Figure 4:**
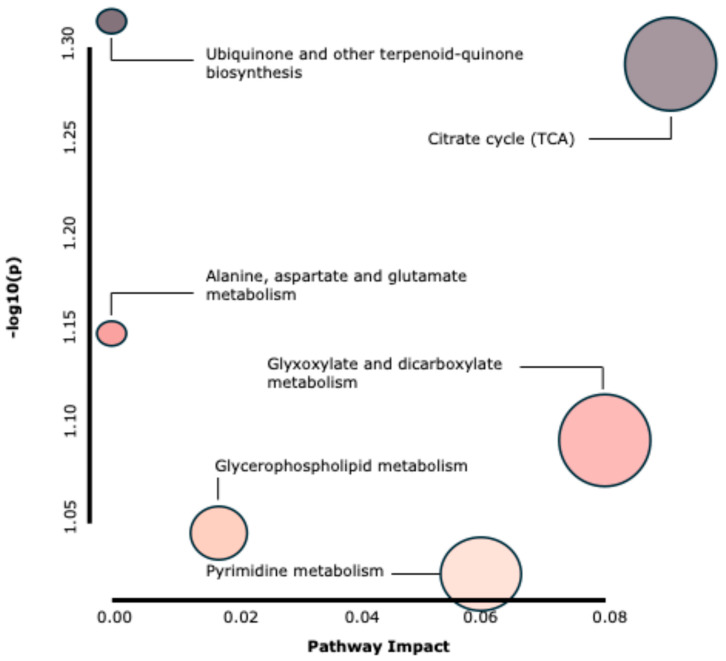
**Overview of the pathway analysis plot.** The screen provides a graphical summary of the pathway analysis in growing rabbits (*Oryctolagus cuniculus*). In the plot, all matched pathways are represented as circles. The color and size of each circle correspond to its *p*-value and pathway impact value, respectively.

Figure 4 shows the pathway analysis plot, which aligns with the graphical summary of the analysis. In this figure, each matched pathway is depicted as a circle, highlighting Ubiquinone and other terpenoid-quinone biosynthesis and Citrate cycle (TCA) metabolism as the most enriched pathways.

The average values related to the validation of the PLS models were R^2^ = 0.89 and Q^2^ = 0.46.

## 3. Discussion

This study aimed to elucidate the effects of diet, feeding systems, and their interactions on metabolic phenotypes, with the aim of identifying potential biomarkers that could inform strategies to mitigate environmental pollution. The most noticeable effect was that of sampling time. This effect may be due to both the level of intake and the possible ‘masking’ effect of cecotrophy, since cecotropes are rich in essential AAs such as lysine, methionine, and threonine, which are crucial for growth and tissue repair. Their re-ingestion ensures efficient protein utilization and supports overall metabolic functions in rabbits. Some of the amino acids that have been observed in higher amounts are the sulfur-containing ones. Regarding intake, although individual intake was not analyzed in this experiment, previous studies observed a significant effect, with a higher intake (+81%; *p* < 0.001) during blood sampling at 21:00 in experiments with similar experimental design [22,23]. Changes in the metabolome due to feed intake have been previously observed in other species such as pigs [24] or broilers [25,26], as well as in farming rabbits [17]. This will be discussed in more detail with specific metabolites, 16 of which were significantly different between the experimental groups.

Firstly, to validate the metabolomics analysis, R^2^ is used to assess the goodness of fit between the model and the observed data, while Q^2^ assesses its predictive ability. According to the SIMCA user manual, a Q^2^ value greater than 0.5 indicates good predictability [27]. Based on the average R^2^ and Q^2^ values obtained in our analysis (0.89 and 0.46, respectively), we conclude that the models have a good fit and good predictive power.

No significant differences were observed when analyzing only the effect of diet over all sampling times. However, when the effect of diet was considered at the different time points (08:00 and 21:00), a better differentiation was obtained, although with greater overlap at 08:00. This could be due to the masking effect of caecotrophy [28,29], which varies throughout the day [30], with caecotroph intake being higher in the morning [31,32]. Since caecotrophs—soft feces rich in essential AAs, which rabbits re-ingest—are an important source of nutrients, it is reasonable to assume that metabolic differences between diets would be smaller in samples taken at 08:00. This is likely to be due to reduced feed intake overnight and increased reliance on cecotrophy [33]. This hypothesis is supported by the fact that the greatest differences were observed at 21:00, where there was no overlap between the experimental diets.

The next step is to define the metabolites that had the most significant impact in terms of quantity—sampling time. As shown in Figure 4, one of the metabolic pathways with the greatest impact and significance is the citrate cycle (TCA). One of the most important metabolites in this cycle is citric acid [34], which is the starting point of this metabolic pathway. There are several experiments in which this metabolite has been studied in rabbits, ranging from studies using it as an additive to studies analyzing it from a metabolic perspective [35]. In our experiment, we observed higher levels of this metabolite at 08:00 (+54%; *p* = 0.0018), a time associated with lower feed intake. This suggests that reduced intake may result in less efficient nutrient utilization, leading to higher circulating levels of the metabolite. Conversely, higher feed intake may increase metabolic efficiency. Marín-García et al. (2024) have previously observed changes in the metabolome of animals influenced by feeding levels. In this case, ascorbic acid, could be used as an indicator of feeding level, as it was significantly reduced (−600%; *p* = 0.001) when animals were subjected to feed restriction [18]. The data presented in this study further support the notion that the TCA metabolic pathway is clearly affected by feeding level, and therefore, some of the metabolites involved may serve as biomarkers of reduced feed intake [36]. During dietary restriction, fatty acid oxidation, AA catabolism, and acetyl-CoA production increase, promoting the regeneration of key metabolites involved in energy production. This increase in metabolic activity may be related to the elevation of certain metabolites that act as antioxidants and regulate urinary pH, possibly as an adaptive response to feed restriction [36,37]. Furthermore, the loss of ascorbic acid may be more closely related to protein restriction rather than energy restriction, suggesting a physiological adaptation to reduced nutrient intake [38].

In this experiment, animals with a higher feed intake had significantly increased *p*-cresol sulfate levels (+353%; *p* = 0.0122) compared to those with a lower intake. Although not statistically significant, a similar study [18] reported an opposite trend. Levels of p-cresol sulfate have been associated with increased intestinal transit time in certain species [39]. It is well known that dietary restriction alters gastrointestinal transit in young rabbits. Gidenne and Feugier [40] observed an increase in mean particle retention time in feed-restricted animals compared to those ad libitum fed. Prolonged colonic transit time enhances bacterial fermentation of amino acids [41,42], which may lead to an overgrowth of proteolytic bacteria in the colon and to alter p-cresol levels, as has been observed in in vitro studies [43].

Regarding the effect of the diet, several significant differences were observed in relation to the lysophosphatidylcholines (LPCs; Table 2, Table 3 and Table 4). LPCs belong to a family of phospholipids and they may be associated with lipid and protein metabolism [44,45]. LPCs metabolites are linked to pantothenic acid in some pathways, another potentially interesting biomarker for future research. Uridine is a compound belonging to the pyrimidine nucleosides class. It is a nucleoside made up of uracil and D-ribose and is a key component of RNA [46]. Pseudouridine is another nucleoside, classified as a nucleoside analog, and is related to uridine. It is formed by post-transcriptional isomerization of specific uridine residues in RNA [47,48]. There are not many studies investigating this metabolite in rabbits. In our case, a higher level of uridine was observed at 21:00 in the experimental diet that is considered to be balanced, Met+ (+118%; *p* = 0.0068). This may be due to the fact that a well-adapted diet is expected to require less de novo nucleotides, which could help to maintain more stable levels of this metabolite and potentially optimize the recycling of these compounds.

One of the most relevant metabolites that appears to discriminate between the dietary protein quality is enterolactone sulfate. Initially, this metabolite is significantly influenced by the sampling time, with higher levels observed in animals fed at 08:00 (+67%; *p* = 0.0003) compared to those who were fed at 21:00. More interestingly, animals fed the Met+ diet had higher enterolactone sulfate concentrations than those fed the Met– diet at both 08:00 (+31%; *p* = 0.0394) and 21:00 (+85%; *p* = 0.0066). The persistence of these differences across at both time points highlights enterolactone sulfate as a promising biomarker for discriminating protein quality. Enterolactone sulfate belongs to the lignan compounds [49]. In other experiments conducted by our research group, we have found that enterolactone is influenced by both diet and genetic background in the same animal species [18]. Several studies have suggested a relationship between enterolactone and changes in bacterial communities, including a reduction in the abundance of certain species and changes in enzymatic activities. Therefore, amino acid modulation appears to have effects on the gut microbiota, positioning this metabolite as a key biomarker in the pursuit of the ideal protein concept.

This study provides insights into metabolic alterations caused by methionine limitation in growing rabbits, though results may be influenced by controlled feeding conditions and specific sampling times, which affect metabolomic profiles. These factors represent limitations when extrapolating findings to other species or natural settings. Nonetheless, identified metabolites such as pseudouridine, citric acid, pantothenic acid, and enterolactone sulfate offer promising targets to refine the ideal protein concept, enhancing nutrient efficiency and reducing environmental impact. Furthermore, applying untargeted metabolomics in wild rabbit nutrition could improve understanding of amino acid requirements in natural populations, supporting conservation and sustainable management strategies, and increasing its populations [50,51,52].

## 4. Materials and Methods

### 4.1. Animal Ethics Statement

The experimental protocols were approved by the Animal Welfare Ethics Committee of the Universitat Politècnica de València (authorisation code: 2018/VSC/PEA/0116) carried out following the Spanish Royal Decree 53/2013 on the protection of animals used for scientific purposes [53]. We have complied with all relevant ethical regulations for animal use.

### 4.2. Experimental Diets

The experimental diets (household) were formulated and pelleted from the same basal mixture and were introduced one day before sampling in order to avoid adaptation mechanisms. The experimental diets were formulated according to the recommendations for growing rabbits in terms of crude protein (155 g/kg dry matter (DM)) and digestible energy (9.86 MJ/kg DM) [54]. Diet was a milled mixture of feedstuffs. The chemical composition of the diets was identical except for the sulfur AA level (Met group: Methionine + Cysteine), which varied in amount by modifying the dietary level of synthetic DL-Methionine. The DL-Methionine supplement was added directly to the diet, and homogenized before granulation. A first Met+ diet had a sulfur AA level of 6.6 g/Kg DM, which had previously been shown to enhance productive traits in a previous study [13]. In a second Met− diet, it was ensured that the sulfur AA level was clearly limited (4.9 g/Kg DM) [13,14,55], by reducing the AA levels by 25%. The variable methionine levels were estimated based on prior research that analyzed the expected amount of this amino acid to be retained in the same genetic type, assuming it behaved similarly to other genetic lines. The ingredients of the basal diet and the chemical composition of the experimental diets are summarized in Table 4.

### 4.3. Experimental Design

**Figure 5:**
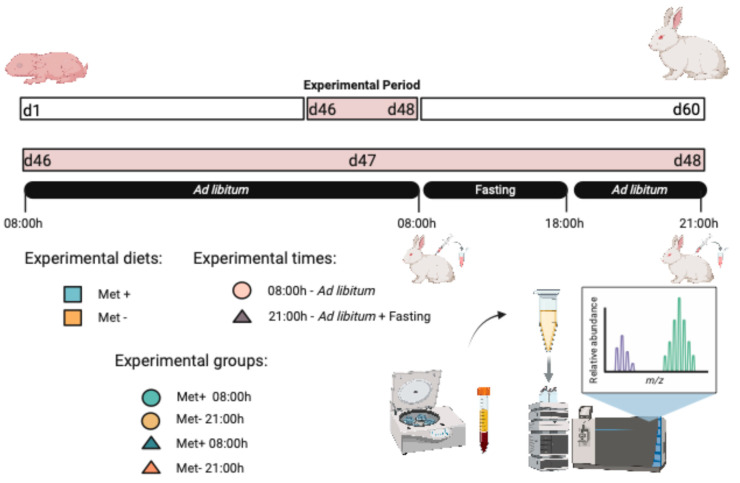
Summary of the experimental design.

The experimental design is shown in Figure 5. A total of 64 three-way (32 males and 32 females) crossbred growing rabbits (H × LP females inseminated with pooled semen from R bucks; H, LP and R lines from the Universitat Politècnica de València, Valencia, Spain) weaned at 28 days of age were used. After weaning, the animals were randomly housed in collective cages (26 × 50 × 31 cm) at a controlled temperature of 10–22 °C throughout the experimental period, under a 12 h light/12 h dark photoperiod (06:00–18:00 light), and fed ad libitum with a commercial diet containing 35 ppm valnemulin and 250 ppm neomycin. On day 46 of age, rabbits were randomly assigned to one of the experimental diets (Met+: *n* = 34 and Met−: *n* = 30). After a one-day of acclimation period (at 08:00 on day 47), a blood sample was extracted from randomly selected animals (*n* = 44), blood samples were taken from the central ear artery (1 mL in EDTA), all blood samples were immediately centrifuged at 700 g for five minutes and the resulting plasma was frozen at −20 °C until further analysis. Following this initial sampling, animals were fasted for 10 h before being refed at 18:00. A second blood sample was then collected at 21:00, three hours after refeeding. This feeding protocol was implemented to optimize, standardize, and synchronize feed intake in growing rabbits after a period of feed deprivation, with the aim of promoting uniform growth, enhancing digestive efficiency, and minimizing variability in nutritional responses among individuals [14]. After the second blood collection, the experimental diet was replaced by the same commercial diet until the end of the growing period (day 60).

### 4.4. Chemical Analysis

Chemical analyses of diets were carried out according to the Association of Official Agricultural Chemists’ methods [56]: 934.01 for DM, 942.05 for ash, 976.06 for crude protein and 920.39 with previous acid hydrolysis of samples for ether extract. Starch content was determined by a two-step enzymatic procedure involving solubilisation and hydrolysis to maltodextrins with thermostable α-amylase, followed by complete hydrolysis with amyloglucosidase (both enzymes from Sigma-Aldrich, Steinheim, Germany) [57], and the resulting glucose was measured by the hexokinase/glucose-6-phosphate dehydrogenase/NADP system (R-Biopharm, Darmstadt, Germany). Neutral detergent fiber, acid detergent fiber and acid detergent lignin were analyzed sequentially [58] according to described methods [56,59], respectively, with a thermostable α-amylase pretreatment and expressed exclusive of residual ash, using a nylon filter bag system (Ankom, Macedon, NY, USA). The AA content of the diets was determined after acid hydrolysis with HCl 6N at 110 °C for 23 h, as previously described [60], using a Waters high-performance liquid chromatography system (Milford, MA, USA). Aminobutyric acid was added as an internal standard after hydrolysis. AAs were derivatized with 6-aminoquinolyl-N-hydroxysuccinimidyl carbamate and separated on a C-18 reverse-phase column (Waters AccQ-Tag, 150 × 3.9 mm). Methionine and cystine were determined separately as methionine sulphone and cysteic acid, respectively, after performic acid oxidation followed by acid hydrolysis.

A total of 88 randomly selected blood samples were submitted for PUN determination. PUN was determined using a commercial kit (Urea/BUN-Color, BioSystems S.A., Barcelona, Spain). The samples were thawed and tempered, after which 1 μL was pipetted into test tubes (a standard and a blank were included in each batch). Then 1 mL of reagent A (sodium salicylate 62 mmol/L, sodium nitroprusside 3.4 mmol/L, phosphate buffer 20 mmol/L and urease 500 U/mL) was added to each sample, mixed thoroughly and incubated at 37 °C for 5 min. Then 1 mL of reactant B (sodium hypochlorite 7 mmol/L and sodium hydroxide 150 mmol/L) was added, mixed thoroughly and incubated for a further 5 min at 37 °C. The absorbance of each sample was then read at 600 nm against the blank.

### 4.5. LC-MS Metabolomics Analysis of Plasma

#### 4.5.1. Chemical Solvents and Standards for Metabolomics Analysis

A total of 68 randomly selected blood samples were analyzed by untargeted metabolomics. High-performance liquid chromatography (HPLC)-grade solvents and eluents were used for the untargeted metabolomics analysis as follows: HPLC-grade acetonitrile (VWR, West Chester, PA, USA), formic acid (FA, Merck KGaA, Darmstadt, Germany) and Milli-Q grade water. Internal standards included during the sample preparation were glycocholic acid (Glycine-1-^13^C), and 4-chloro-DL-phenylalanine (Merck KGaA, Darmstadt, Germany).

#### 4.5.2. Sample Preparation and LC-MS Analysis

Plasma from each animal was analyzed separately. Plasma was prepared by deproteinisation of 150 µL of sample with 450 µL of ice-cold acetonitrile (100% acetonitrile [ACN]) containing an internal standard mix of glycocholic acid (glycine-1-^13^C) and p-chlorophenylalanine at a final concentration of 0.01 mg/mL. Samples were prepared in 96-well plates with 1 mL wells. Plates were mixed for 1 min, incubated at 4 °C for 10 min, and centrifuged at 2250 g for 25 min at 4 °C. Approximately 400 µL of supernatant was transferred to Phenomenex 96-square well filter plates. Vacuum was applied to the plates and the solvent containing plasma metabolites was collected in a collection plate. The filtered supernatant was transferred to two 200 µL 96-well plates (65 µL per well) and the plates were vacuum centrifuged to dryness (ca. 2.5 h, 805× *g* at 30 °C). Samples were resuspended in a mix of H_2_O:ACN:FA (95:5:0.1) using the same volume as before evaporation. A protective film was welded onto the plate using a heat sealer, and the plates were centrifuged at 2250× *g*, 4 °C for 25 min before the LC-MS analysis.

The samples were analyzed by Ultra High-Performance Liquid Chromatography (UHPLC) using a Nexera X2 LC coupled to a LCMS-9030 Q-TOF MS system (Shimadzu Corporation, Kyoto, Japan) using negative electrospray ionization (ESI). Chromatographic separations were performed on an Acquity HSS T3 column (1.7 µm, 100 × 2.1 mm, Waters Ltd., Elstree, UK). The column temperature was set to 40 °C, the samples were held at 10 °C and 3 µL aliquots were injected onto the column. The chromatographic system used a binary gradient of Solvent A (water with 0.1% formic acid) and Solvent B (acetonitrile with 0.1% formic acid) at a flow rate of 0.4 mL/min. A linear gradient was used from 5% B to 100% B over 12 min, with a hold at 100% for 1 min before returning to the initial conditions of 5% B for 3 min for column re-equilibration. This resulted in a total analysis time per sample of 16 min. Qualitative untargeted analysis of the plasma metabolome was performed in negative mode. Data were collected in full scan (MS) and autoMS/MS modes from 50 to 1000 *m/z*. The following MS parameters were used: ion-source temperature, 300 °C; heated capillary temperature, 250 °C; heating block temperature, 400 °C; electrospray voltage −3.5 kV (ESI-); electrospray nebulisation gas flow, 3 L/min; drying gas flow, 10 L/min; detector voltage, 2.02 kV. MS/MS spectra of the QC samples were obtained using data dependent acquisition (DDA) with a MS TOF scan followed by 10 dependent events, WHERE precursor ions continuously selected for fragmentation during the run. Precursor ions were fragmented at 20 eV collision energy (CE) with a ±10 eV CE spread. Mass calibration was performed externally using a sodium iodide solution (400 ppm in methanol) from *m/z* 50–1000. Data acquisition was performed using LabSolutions software version 5.114 (Shimadzu Corporation, Kyoto, Japan).

#### 4.5.3. Sample Quality Control and Metabolomics Data Pre-Processing

Quality control samples (QCs) were used to monitor the quality of the chromatographic runs, the UPLC system stability and the accuracy of sample preparation. Plasma QCs were prepared by pooling an aliquot of all samples and subjecting this pooled sample to the same sample preparation protocol as the samples. The QCs were injected several times throughout the analysis as well as at the beginning and end of the analysis, and were used in the data pre-processing for signal drift correction. Blanks were injected during the chromatographic analysis to monitor for external contamination from solvents, eluents and carry-over effects. The sample order for the chromatographic analysis was randomized to eliminate bias in the results and to ensure that each sample group was equally affected.

MS-DIAL software (Version 5.4.241021) was used to perform peak detection, alignment and gap filling for the data files, with the option for identification of metabolites using the predefined library of this tool. The MS-DIAL generated data matrix was exported to Excel and filtered to eliminate peaks present in blanks, and retention time was truncated to contain only portions containing chromatographic peaks, while masses above 700 *m/z* were discarded.

An initial principal component analysis (PCA) was performed using Metaboanalyst to check the quality of the dataset and to eliminate potential outliers. Partial least-squares discriminant analysis (PLS-DA) models were built to identify the metabolites responsible for the differences between the experimental diets. Validation of the models was performed using repeated random subsampling validation. Models were assessed using the explained variation in Y, plots depicting actual and predicted values, and the proportion of variation explained (R2). Variables for identification were selected using variable importance in projection (VIP) scores. To validate the PLS-DA models obtained, a cross-validation was performed using 5 as the maximum number of components to search, along with the 5-fold CV method. Both R^2^ and Q^2^ were calculated for each generated PLS model.

#### 4.5.4. Metabolite Identification

Metabolites were identified based on queries in the Human Metabolome Database (http://www.hmdb.ca, accessed on 7 May 2025) online database to obtain possible chemical structures using accurate mass and mass spectrometric fragmentation patterns.

#### 4.5.5. Metabolites Statistical Analysis

The identified metabolites were statistically analyzed using the GLM procedure of SAS (2002). The model included as fixed effects the animal (each of the selected animals becomes a block), the sampling time (8:00 or 21:00) and the experimental diet (Met+ and Met−), as well as the interaction.

Least-square means were obtained with their standard errors and compared using t-test, with significance level set at *p* < 0.05. Orthogonal contrasts between experimental diets were also performed (*t*-test).

## 5. Conclusions

This work shows that untargeted metabolomics is indeed sensitive to a diet with limited AA and different sampling periods. A clear ‘compensatory’ effect produced by cecotrophy is observed, indicating an adaptive advantage of the species in terms of protein nutrition. This study suggests that pseudourine, citric acid, pantothenic acid and enterolactone sulfate are potential biomarkers that allow for a deeper understanding of the concept of ideal protein and for mitigating the environmental impact effects of livestock farming.

## Figures and Tables

**Table 1:** List of plasma metabolites differentiating among sampling time (*) in growing rabbits (*n* = 54).

			08:00 h		21:00 h			
RT-*m/z*	ION	Metabolite **	X	SE	X	SE	Fold	*p*-Value
1.05/243.0655	[M-H]-	Pseudouridine	3832	±148	3414	±193	1.122	0.0926
1.30/191.0235	[M-H]-	Citric acid	79,074	±5111	51,472	±6664	1.536	0.0018
2.00/243.0670	[M-H]-	Uridine	7559	±772	5592	±1006	1.351	0.1271
3.20/218.1077	[M-H]-	Panthothenic acid	5469	±196	4412	±256	1.240	0.0019
3.99/212.0073	[M-H]-	Indoxylsulfate	4270	±684	8175	±892	0.522	0.0011
4.13/178.0550	[M-H]-	Hippuric acid	44,145	±1561	39,954	±2036	1.105	0.1083
5.38/187.1013	[M-H]-	Azelaic acid	12,267	±1057	7407	±1379	1.656	0.0072
5.68/137.0274	[M-H]-	4-Hydroxybenzoic acid	6990	±487	10,529	±634	0.664	<0.0001
6.94/329.2392	[M-H]-	Octadecenoic acid ***	97,105	±21,496	28,878	±28,027	3.363	0.0589
7.23/229.1472	[M-H]-	Dodecanedioic acid	7407	±469	4837	±611	1.531	0.0016
10.36/566.3580	[M+HCOO]-	LysoPC(18:1(11Z)/0:0)	168,409	±8863	116,908	±11,557	1.441	0.0009
8.57/391.2913	[M-H]-	Deoxycholic acid	9823	±579	6434	±754	1.527	0.0008
10.17/295.2344	[M-H]-	9-Hydroxylinoleic acid	15,357	±1517	4008	±1978	3.832	<0.001
9.64/564.3369	[M-H]-	LysoPC(18:2/0:0)	304,386	±13,309	227,654	±17,353	1.337	0.0009
9.64/564.4826	[M-H]-	LysoPC	2787	±411	4445	±536	0.623	0.0175
9.07/562.3211	[M+HCOO]-	LysoPC(18:3(9Z,12Z,15Z)/0:0)	14,797	±812	9477	±1059	1.561	0.0002
8.01/407.2851	[M-H]-	Allocholic acid	14,915	±1598	2135	±2084	6.986	<0.0001
11.25/568.3632	[M+Fa-H]-	LysoPC(18:0/0:0)	263,130	±12,369	214,175	±16,127	1.229	0.0196
4.58/187.0108	[M-H]-	P-cresol sulfate	4350	±2583	15,376	±3368	0.283	0.0122
11.25/508.3527	[M-H]-	LysoPC(18:0/0:0)—Fragment	61,711	±3043	47,903	±3967	1.288	0.0079
5.85/377.0747	[M-H]-	Enterolactone sulfate	14,640	±920	8749	±1200	1.673	0.0003
0.66/92.9288	[M-H]-	Chloroacetic acid	7160	±53	6664	±69	1.074	<0.0001
9.18/311.2288	[M-H]-	11-HpODE ****	13,833	±3042	3091	±3966	4.475	0.0363

* At 08:00 h, after 24 h of ad libitum access to the experimental diet, and at 21:00 h, 3 h after refeeding following a 10 h fasting. ** Identification was performed tentatively using MS/MS and METLIN/HMDB databases. *** 5,8,12-Trihydroxy-9-octadecenoic acid. **** (11S)-11-hydroperoxylinoleic acid.

**Table 2:** List of plasma metabolites differentiating among experimental diets in growing rabbits at 08:00 h (*n* = 34).

			Met+		Met−			
RT-*m*/*z*	ION	Metabolite *	X	SE	X	SE	Fold	*p*-Value
1.05/243.0655	[M-H]-	Pseudouridine	3726	±215	3926	±203	0.949	0.5043
1.30/191.0235	[M-H]-	Citric acid	72,695	±7373	84,743	±6952	0.858	0.2432
2.00/243.0670	[M-H]-	Uridine	7543	±1219	7574	±1150	0.996	0.9857
3.20/218.1077	[M-H]-	Panthothenic acid	5434	±274	5501	±258	0.988	0.8599
3.99/212.0073	[M-H]-	Indoxylsulfate	4767	±746	3829	±703	1.245	0.3669
4.13/178.0550	[M-H]-	Hippuric acid	45,038	±1972	43,353	±1860	1.039	0.5384
5.38/187.1013	[M-H]-	Azelaic acid	12,329	±1909	12,213	±1800	1.009	0.9650
5.68/137.0274	[M-H]-	4-Hydroxybenzoic acid	7252	±669	6758	±632	1.073	0.5984
6.94/329.2392	[M-H]-	Octadecenoic acid **	86,309	±37,309	106,702	±35,175	0.809	0.6935
7.23/229.1472	[M-H]-	Dodecanedioic acid	8083	±733	6808	±691	1.187	0.2147
10.36/566.3580	[M+HCOO]-	LysoPC(18:1(11Z)/0:0)	157,480	±14,462	178,124	±13,635	0.884	0.3068
8.57/391.2913	[M-H]-	Deoxycholic acid	9689	±964	9943	±909	0.974	0.8493
10.17/295.2344	[M-H]-	9-Hydroxylinoleic acid	14,240	±2670	17,238	±2545	0.826	0.2893
9.64/564.3369	[M-H]-	LysoPC(18:2/0:0)	287,263	±21,681	319,608	±20,441	0.899	0.2858
9.64/564.4826	[M-H]-	LysoPC	4328	±490	1418	±462	3.052	0.0001
9.07/562.3211	[M+HCOO]-	LysoPC(18:3(9Z,12Z,15Z)/0:0)	13,964	±1381	15,538	±1303	0.899	0.4137
8.01/407.2851	[M-H]-	Allocholic acid	15,682	±2957	14,281	±2787	1.098	0.7423
11.25/568.3632	[M+Fa-H]-	LysoPC(18:0/0:0)	238,330	±20,434	285,174	±19,265	0.836	0.1051
4.58/187.0108	[M-H]-	P-cresol sulfate	4008	±1584	4654	±1493	0.861	0.7685
11.25/508.3527	[M-H]-	LysoPC(18:0/0:0)—Fragment	55,592	±4856	67,151	±4579	0.828	0.0929
5.85/377.0747	[M-H]-	Enterolactone sulfate	16,751	±1351	12,763	±1274	1.312	0.0394
0.66/92.9288	[M-H]-	Chloroacetic acid	7055	±31	7253	±29	0.973	<0.001
9.18/311.2288	[M-H]-	11-HpODE ***	12,554	±5395	14,969	±5086	0.839	0.7467

* Identification was performed tentatively using MS/MS and METLIN/HMDB databases. ** 5,8,12-Trihydroxy-9-octadecenoic acid; *** (11S)-11-hydroperoxylinoleic acid.

**Table 3:** List of plasma metabolites differentiating among experimental diets in growing rabbits at 21:00 h (*n* = 20).

			Met+		Met−			
RT-*m*/*z*	ION	Metabolite *	X	SE	X	SE	Fold	*p*-Value
1.05/243.0655	[M-H]-	Pseudouridine	3405	±372	3418	±243	0.996	0.9762
1.30/191.0235	[M-H]-	Citric acid	40,552	±12,211	56,153	±7994	0.722	0.2992
2.00/243.0670	[M-H]-	Uridine	9008	±1335	4129	±874	2.182	0.0068
3.20/218.1077	[M-H]-	Panthothenic acid	4959	±501	41,777	±328	0.119	0.2087
3.99/212.0073	[M-H]-	Indoxylsulfate	9418	±2200	7642	±1440	1.232	0.5080
4.13/178.0550	[M-H]-	Hippuric acid	45,581	±4327	37,541	±2833	1.214	0.1375
5.38/187.1013	[M-H]-	Azelaic acid	8137	±70,983	7093	±647	1.147	0.3884
5.68/137.0274	[M-H]-	4-Hydroxybenzoic acid	12,399	±1210	9728	±792	1.275	0.0813
6.94/329.2392	[M-H]-	Octadecenoic acid **	8914	±30,025	37,435	±19,656	0.238	0.4371
7.23/229.1472	[M-H]-	Dodecanedioic acid	6075	±895	4306	±586	1.411	0.1157
10.36/566.3580	[M+HCOO]-	LysoPC(18:1(11Z)/0:0)	139,923	±14,803	99,346	±7634	0.131	0.0083
8.57/391.2913	[M-H]-	Deoxycholic acid	7883	±951	5813	±623	1.356	0.0854
10.17/295.2344	[M-H]-	9-Hydroxylinoleic acid	4108	±1370	3963	±897	1.037	0.9306
9.64/564.3369	[M-H]-	LysoPC(18:2/0:0)	261,742	±22,293	202,718	±12,460	1.229	0.0164
9.64/564.4826	[M-H]-	LysoPC	965	±389	1583	±255	0.610	0.2010
9.07/562.3211	[M+HCOO]-	LysoPC(18:3(9Z,12Z,15Z)/0:0)	11,709	±1075	8521	±704	1.374	0.0232
8.01/407.2851	[M-H]-	Allocholic acid	2091	±505	2154	±331	0.971	0.9188
11.25/568.3632	[M+Fa-H]-	LysoPC(18:0/0:0)	226,973	±18,439	208,690	±12,071	1.088	0.4176
4.58/187.0108	[M-H]-	P-cresol sulfate	9371	±9720	17,949	±6363	0.522	0.4698
11.25/508.3527	[M-H]-	LysoPC(18:0/0:0)—Fragment	50,689	±5351	46,709	±3503	1.085	0.5416
5.85/377.0747	[M-H]-	Enterolactone sulfate	12,891	±1611	6974	±1055	1.848	0.0066
0.66/92.9288	[M-H]-	Chloroacetic acid	6771	±194	6618	±127	1.023	0.5180
9.18/311.2288	[M-H]-	11-HpODE ***	538	±3542	4186	±2319	0.129	0.4003

* Identification was performed tentatively using MS/MS and METLIN/HMDB databases. ** 5,8,12-Trihydroxy-9-octadecenoic acid *** (11S)-11-hydroperoxylinoleic acid.

**Table 4:** Ingredients (g/Kg) and chemical composition (g/Kg DM) of the experimental diets.

Ingredients		Chemical Composition	Met+	Met−
Wheat gran	300	Dry matter ^2^	907	907
DDGS corn	50	Ash ^2^	104	104
Bakery by-product	30	Crude protein ^2^	155	155
Sunflower meal	36	Crude fat ^2^	29.7	29.7
Alfalfa meal	334	Neutral detergent fiber (NDF) ^2^	455	455
Beet pulp	80	Acid detergent fiber (FAD) ^2^	29.7	29.7
Straw	136	Acid detergent lignin (ADL) ^2^	455	455
Beet molasses	13.9	Digestible energy ^2^	262	262
L-Arginine	3.1	Amino acid composition ^3^:		
L-Histidine	1.5	Aspartic acid	12.95	12.95
Calcium carbonate	6.5	Serine	5.74	5.74
Sodium clorhide	4	Glutamic acid	22.96	22.96
Vitamine/mineral ^1^	5	Glycine	6.53	6.53
		Histidine	3.57	3.57
		Arginine	9.28	9.28
		Threonine	6.90	6.90
		Alanine	6.69	6.69
		Proline	8.04	8.04
		Cystine	2.37	2.37
		Tyrosine	2.90	2.90
		Valine	7.03	7.03
		Methionine ^4^ + Cystine	6.60	4.90
		Isoleucine	5.07	5.07
		Lysine	8.10	8.10
		Leucine	9.70	9.70
		Phenylalanine	5.58	5.58

^1^ Contents per kg of feed: vitamin A: 8375 IU; vitamin D3: 750 IU; vitamin E: 20 mg; vitamin K3: 1 mg; vitamin B1: 1 mg; vitamin B2: 2 mg; vitamin B6: 1 mg; nicotinic acid: 20 mg; choline chloride: 250 mg; magnesium: 290 mg; manganese: 20 mg; zinc: 60 mg; iodine: 1.25 mg; iron: 26 mg; copper: 10 mg; cobalt: 0.7 mg; butyl hydroxyanisole and ethoxyquin mixture: 4 mg. ^2^ Provided by the manufacturer (NANTA, Valencia, Spain). ^3^ Analyzed. ^4^ Levels of this amino acid vary depending on experimental diets, values were obtained by adding DL-methionine.

## Data Availability

Pablo Jesús Marín-García should be contacted to request data from this study.

## Abbreviations

AA	Amino acid
LC−MS	liquid chromatography−mass spectrometry
PCA	principal component analysis
PLS-DA	partial least-squares discriminant analysis
ACN	acetonitrile
IS	internal standards
HPLC	high-performance liquid chromatography
*m*/*z*	mass to charge ratio
QC	quality control
LPCs	lysophosphatidylcholines
